# Simultaneous occurrence of a severe Morel-Lavallée lesion and gluteal muscle necrosis as a sequela of transcatheter angiographic embolization following pelvic fracture: a case report

**DOI:** 10.1186/s13256-015-0550-7

**Published:** 2015-03-26

**Authors:** Takayoshi Shimizu, Shuichi Matsuda, Atsushi Sakuragi, Tomio Tsukie, Keiichi Kawanabe

**Affiliations:** 1 Department of Orthopedic Surgery, Graduate School of Medicine, Kyoto University, 54 Kawaharacho, Syogoin, Sakyo-ku, Kyoto 606-8507 Japan; 2 Department of Orthopedic Surgery, Nagahama City Hospital, 313 Ouinuicho, Nagahama, Shiga 526-0043 Japan; 3 Department of Plastic Surgery, Kitano Hospital, 2-4-20 Ougimachi, Kita-ku, Osaka city, Osaka 530-8480 Japan; 4 Department of Orthopedic Surgery, Shiga Medical Center for Adults, 5-4-30 Moriyama, Moriyama city, Shiga 524-8524 Japan

**Keywords:** Fillet flap, Gluteal muscle necrosis, Hemipelvectomy, Morel-Lavallée lesion, Pelvic fracture, Sepsis, Transcatheter angiographic embolization

## Abstract

**Introduction:**

Morel-Lavallée lesions are posttraumatic hemolymphatic collections caused by disruption of the interfascial planes between the subcutaneous soft tissue and muscle. Severe peripelvic Morel-Lavallée lesions have rarely been reported in the literature. By contrast, a number of cases of gluteal muscle necrosis following transcatheter angiographic embolization for pelvic fracture have been reported. Each entity can result in severe infection and sepsis, and the mortality rate in such cases is quite high. However, to date, no previous reports have described a case in which these life-threatening entities occurred simultaneously.

**Case presentation:**

A 32-year-old Asian man simultaneously developed severe peripelvic Morel-Lavallée lesions and gluteal muscle necrosis with sepsis following transcatheter angiographic embolization after an unstable pelvic fracture. Extremely large skin and soft tissue defects, which were untreatable with any commonly used flaps, were generated after repeated debridement. In addition, a deep-bone infection was suspected in his left fractured iliac bone, while motor function was almost completely lost in his left leg, possibly as a sequela of transcatheter angiographic embolization. As a result of his condition, a left hemipelvectomy was unavoidable. A pedicled fillet flap from his sacrificed left limb was used for the treatment of the defects and to provide a durable base for a prosthesis. Our patient survived and returned to his previous job 24 months after the surgery wearing a prosthetic left leg.

**Conclusion:**

As illustrated by the present case, severe peripelvic Morel-Lavallée lesions and gluteal muscle necrosis following transcatheter angiographic embolization can occur simultaneously after unstable pelvic fractures. Physicians should recognize that these entities can result in life-threatening sepsis and, therefore, should attempt to detect them as early as possible. When hemipelvectomy is unavoidable, a pedicled upper and lower leg in-continuity fillet flap may provide satisfactory outcomes.

## Introduction

The Morel-Lavallée lesion was first described in the mid-nineteenth century as a ‘closed degloving injury’ in the proximal thigh [1]. More particularly, the condition was characterized as a traumatic separation and hemolymphatic collection between the subcutaneous soft tissue and muscle. Subsequently, the term has been used in the literature to describe similar lesions in other parts of the body. However, to date, only one previous report has described a large life-threatening Morel-Lavallée lesion complicated with sepsis after pelvic trauma [2].

Unstable pelvic fracture is a critical injury with a reported mortality rate of 6.4% to 30% [3-6]. Margolies *et al.* first described the successful treatment of a patient with this condition using transcatheter angiographic embolization (TAE), and this technique has subsequently proven to be one of the most useful methods for controlling hemorrhage associated with pelvic fracture [7]. However, gluteal muscle necrosis caused by TAE has been reported for cases in which embolization of the bilateral internal iliac arteries was required because of uncontrolled bleeding [8,9]. In particular, Takahira *et al.* reported that five of 151 patients (3.3%) who underwent bilateral iliac TAE for pelvic fracture developed gluteal muscle necrosis [8]. In such cases, the development of sepsis has typically proven fatal. Lower limb paresis as a sequela of TAE of the iliac artery has also been reported [10].

We present here a case of a severe Morel-Lavallée lesion with sepsis in a 32-year-old Asian man after an unstable pelvic fracture. Our patient also had severe gluteal muscle necrosis and left lower limb paresis following TAE. To the best of our knowledge, there have been no previous reports of patients with simultaneous severe Morel-Lavallée lesions and gluteal muscle necrosis as a sequela of TAE. We describe a method for the reconstruction of an extremely large soft-tissue defect that achieved satisfactory outcomes in our case.

## Case presentation

**Figure 1:**
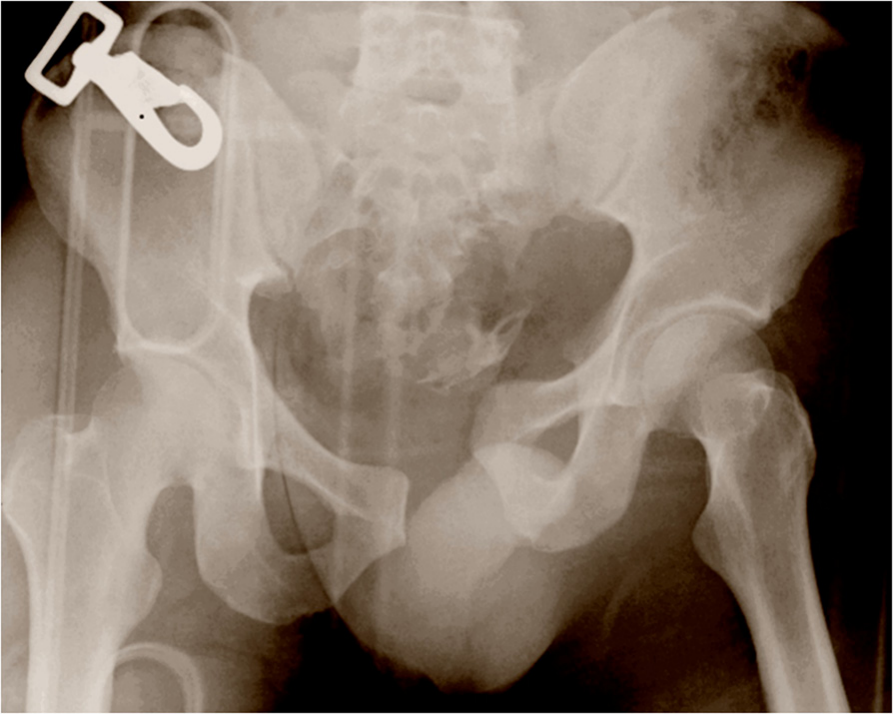
**Anteroposterior X-ray of the pelvis showing an unstable pelvic fracture (AO classification type C1-3).**

A healthy 32-year-old Asian man was involved in a major road traffic accident. On admission, a pelvic X-ray showed an unstable fracture of his left pelvis (AO classification type C1-3) and fractures of the lumbar transverse processes (Figure 1). He was in hemorrhagic shock due to retroperitoneal bleeding. No injuries were observed in his head or chest. An experienced radiologist performed emergency TAE of his internal iliac artery using a hemostatic gelatin sponge (Spongel) to control the bleeding. However, embolization of his bilateral internal iliac arteries was needed to control the bleeding successfully.

**Figure 2:**
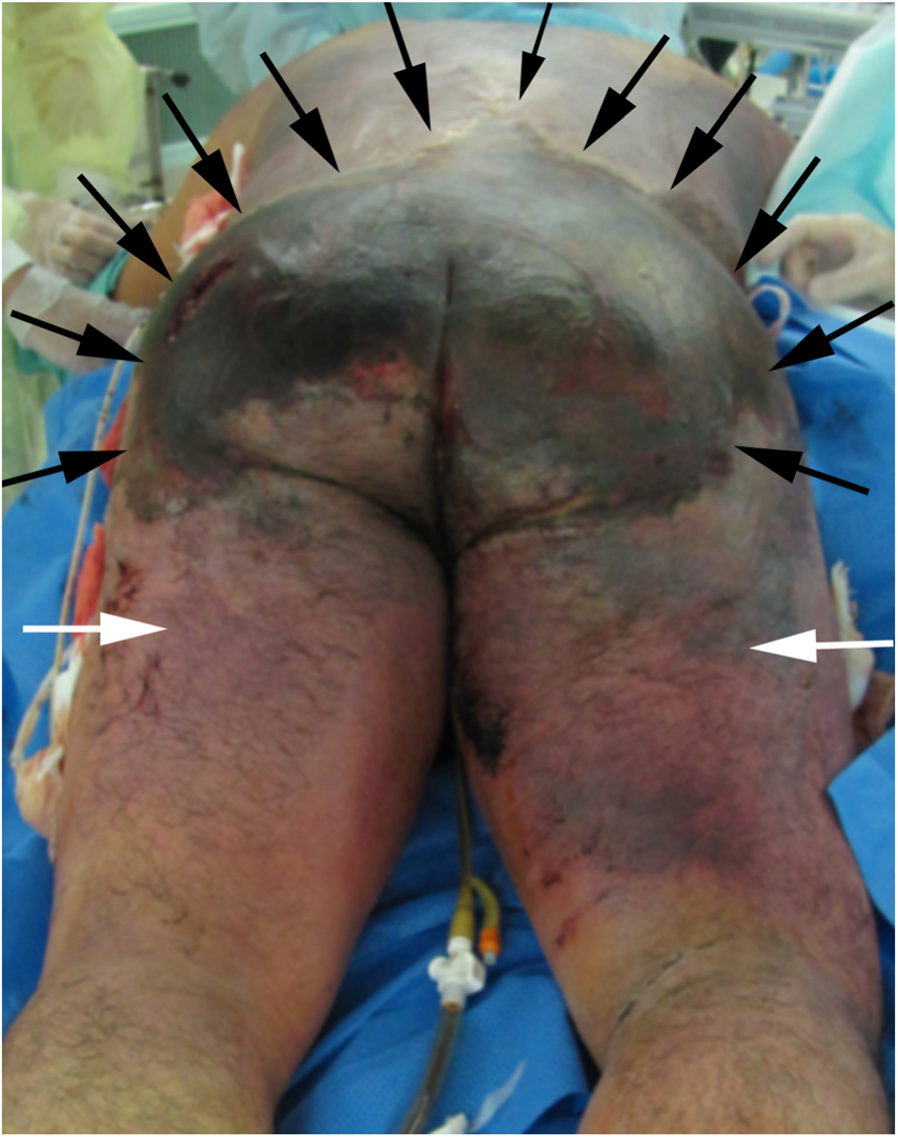
**Necrosis of the back and bilateral buttocks (black arrows) seven days after injury.** The changes in the skin condition extended to the thighs (white arrows).

**Figure 3:**
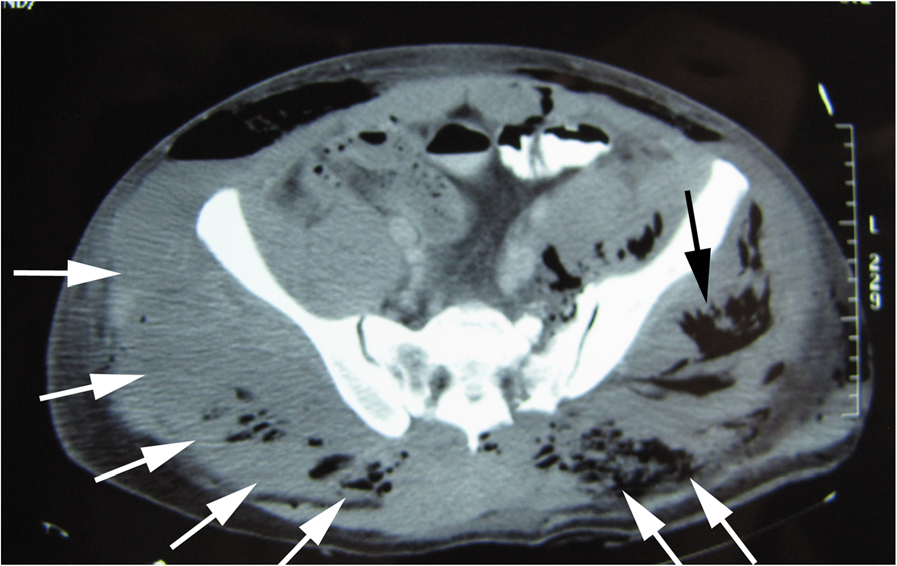
**Computed tomography seven days after injury showed left gluteal muscle damage (black arrow), and a massive bilateral collection of fluid and gas between the gluteal muscles and subcutaneous soft tissue (white arrows).**

**Figure 4:**
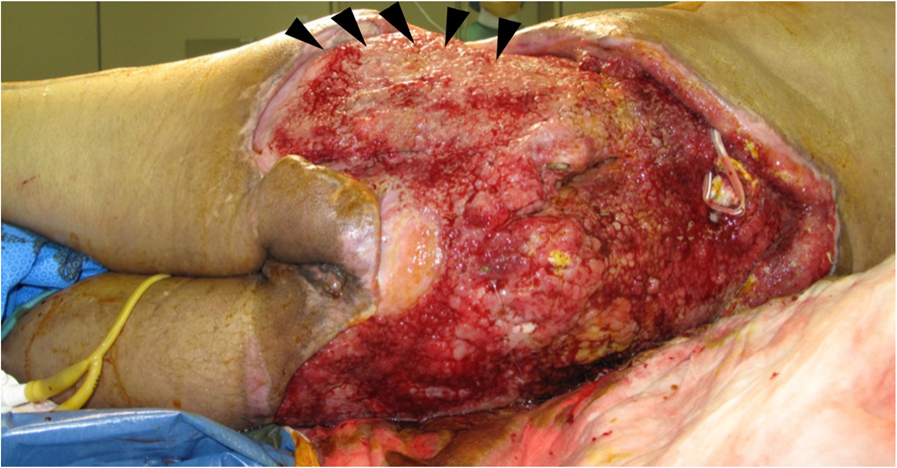
**Extensive defects developed after several debridements.** The site of the left iliac bone fracture is exposed. Deep-bone infection was strongly suspected (black arrowheads).

Over the course of seven days, skin erosions on both buttocks advanced to necrosis (Figure 2). Computed tomography (CT) showed left gluteal muscle damage and a massive bilateral collection of fluid and gas between the gluteal muscles and subcutaneous soft tissue (Figure 3). Our patient also developed sepsis. Emergency surgical debridement was undertaken, and a massive amount of brownish pus was drained from the subcutaneous cavity. His left gluteal muscle seemed to be severely damaged, and some areas of his bilateral gluteal muscles also appeared to be necrotic. A variety of bacteria (*Citrobacter*, *Enterobacter*, *Enterococcus* and *Pseudomonas aeruginosa*) was grown from a culture of the pus. On day 11, a tracheostomy was performed. Several debridements were subsequently performed, and we removed almost all of his bilateral gluteal muscles. This exposed his left fractured iliac bone, which was suspected of having a deep-bone infection (Figure 4). As a result of the debridement, an extensive soft-tissue defect was created in his buttocks and extended into his thighs. Additionally, left lower limb paresis became obvious as our patient’s level of consciousness improved. Both sensory and motor function were almost completely lost in his left leg.

**Figure 5:**
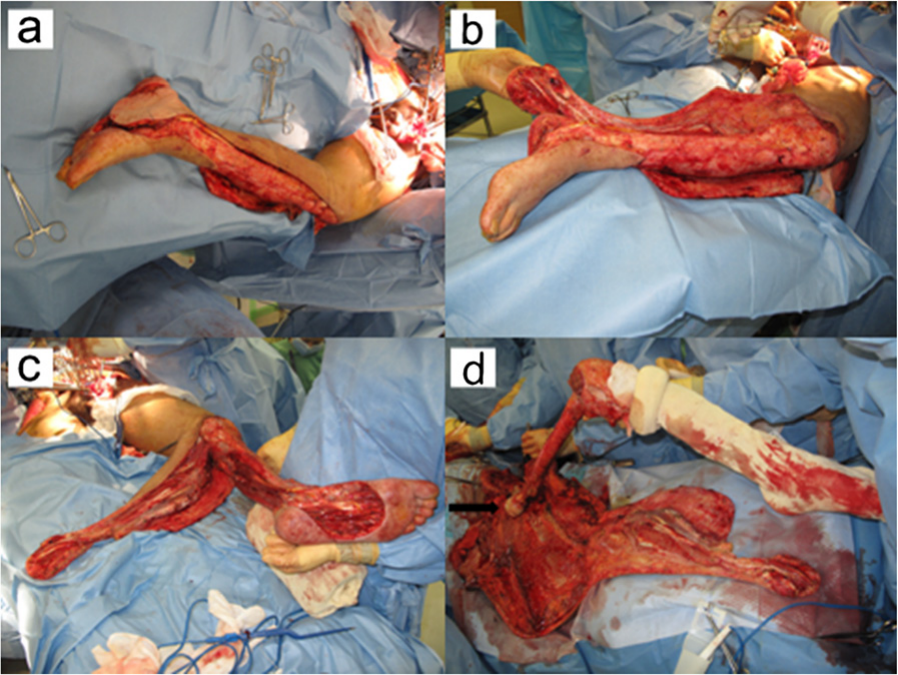
**Intraoperative picture showing the fillet flap. (a-c)** A fillet flap containing all the muscles and skin of the upper leg, gastrocnemius and soleus muscles, and a medial pedis flap were harvested (white arrowheads). **(d)** The femur and all the bones of the lower limb were removed (black arrowhead; femoral head).

**Figure 6:**
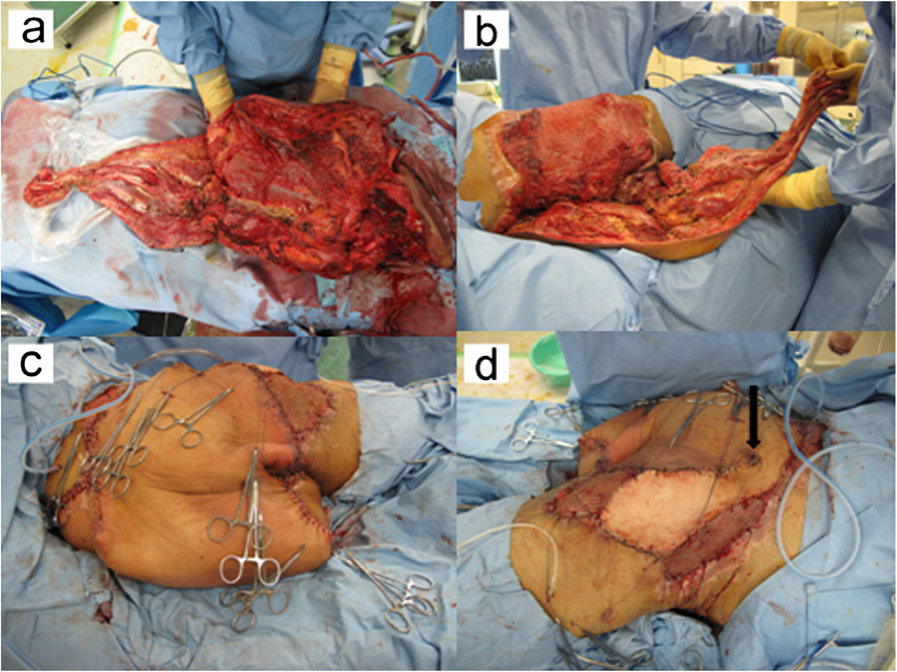
**Intraoperative picture showing the successful coverage of the soft- tissue defect. (a-c)** The bilateral gluteal defects were almost covered using the pedicled fillet flap (black arrowheads). **(d)** Anterior view (black arrow; umbilicus).

Based on our patient’s condition, sacrificing his paralyzed left limb was considered the only option for achieving a satisfactory outcome. In particular, it would allow the extensive soft-tissue defect to be appropriately covered, and good ambulatory function was anticipated based on the use of a prosthesis. Therefore, we suggested hemipelvectomy and the use of a pedicled fillet flap from the sacrificed left limb for treatment of the soft-tissue defect. Our patient and his family agreed to this procedure. On day 95, we performed the surgical procedure with urologists and plastic surgeons, attempting reconstruction with the lower leg pedicled fillet flap subsequent to the hemipelvectomy. An incision was placed to harvest a vascularized fillet flap containing all the muscles and skin of the left upper leg, the gastrocnemius and the soleus muscles, and a medial pedis flap. The pedicle of the flap included the femoral, popliteal and posterior tibial arteries. Next, we performed the left hemipelvectomy, and then removed all of the bones of his left lower limb (Figure 5). Consequently, the bilateral gluteal defects were almost filled using the pedicled fillet flap (Figure 6). The remaining skin defect was covered with a split-thickness skin graft harvested from his amputated lower limb. The operation took 12 hours.

**Figure 7:**
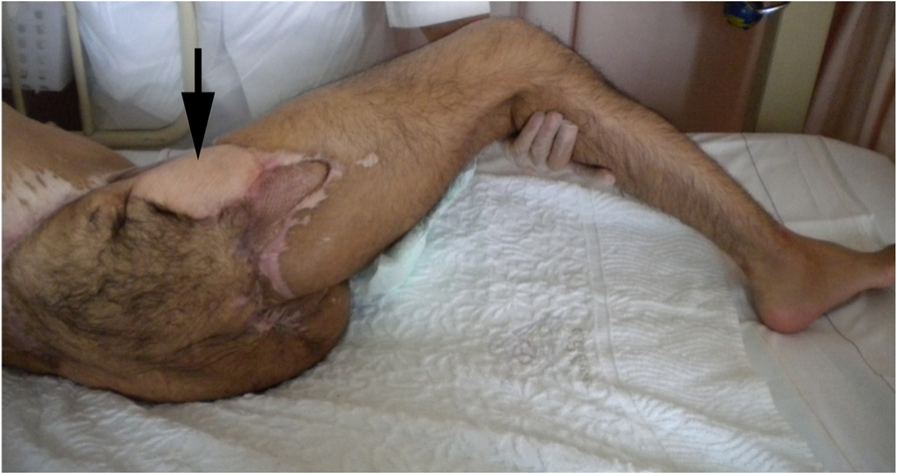
**Complete cure of the wound of the buttocks (black arrow) six months after reconstructive surgery.**

After the operation, several minor surgeries were needed to promote wound-healing. Six months after surgery, the wound was completely healed (Figure **7**), and our patient was transferred to another hospital for rehabilitation. At 24 months post-surgery, he had successfully returned to his previous office work wearing a prosthetic left leg.

## Discussion

In the present case, the pathology underlying the severe peripelvic lesion can be explained by considering three entities: the Morel-Lavallée lesions, the gluteal muscle necrosis following TAE, and direct contusion. In particular, the CT image on the seventh day revealed subcutaneous fluid collection and left gluteal muscle damage, indicating both Morel-Lavallée lesions and direct contusion. The intra-operative findings indicated right gluteal muscle necrosis as a sequela of the TAE. To the best of our knowledge, no previous reports have described a case in which these life-threatening conditions were superimposed.

With respect to the present report, only one case of a severe Morel-Lavallée lesion in the pelvis has been reported in the literature [2]. Phillips *et al.* stated that obesity may increase the risk of developing Morel-Lavallée lesions because it can lead to increased vulnerability of the perforating arteries [2]. In addition, the increased amount of subcutaneous fat may generate a greater shearing force. Because our patient was not obese and did not have any medical history, the seriousness of the lesion was attributed to an extremely high shearing force generated toward both sides of his buttocks. Morel-Lavallée lesions should have been noticed as early as possible, because its early detection and management might have prevented sepsis by controlling the local overgrowth and subsequent dissemination of bacteria. Magnetic resonance imaging (MRI) is considered a reliable modality for describing effusions and determining the lesion type [11]. However, MRI was difficult to perform in our case because of our patient’s state of shock. Ultrasound could be a useful diagnostic tool for detecting the fluid collections and underlying muscle contusion or laceration, as described by some authors, and can be easily performed in patients in shock [12,13]. In light of the present case, an important recognition is that Morel-Lavallée lesions resulting from high-energy injuries can be potentially severe.

Suzuki *et al.* found that initial traumatic contusion of the gluteal muscles tends to contribute to the development of necrosis [14]. MRI or CT may confirm whether subclinical damage to the gluteal muscles is present. If TAE is needed in a case in which a high risk of necrosis is expected based on CT or MRI, selective TAE should be performed [9].

Both Morel-Lavallée lesions and TAE induce the development of peripelvic soft-tissue ischemia and increase the risk of contamination of any hematoma, with subsequent abscess formation and risk of sepsis [15]. In such severe cases, an open approach and extensive debridement should be performed. However, percutaneous drainage with or without sclerotherapy or vacuum-assisted therapy may be indicated in minor cases [16,17]. In our case, frequent daily debridement with pulsatile irrigation in the intensive care unit was needed after the first surgical debridement and open drainage, in conjunction with the administration of broad-spectrum antibiotics. Thus, the successful healing of severe peripelvic soft-tissue infection requires substantial resources in terms of manpower, material and patience.

The use of free flaps (for example, the rectus abdominis musculocutaneous flap, anterolateral thigh flap and latissimus dorsi muscle) and the free fillet lower leg flap have been reported for the reconstruction of extensive defects after hemipelvectomy or exenteration of a malignancy, with the latter type of flap particularly utilized for hemipelvectomy [18-22]. Butler described the efficiency of the pedicled upper and lower leg in-continuity fillet flap for reconstruction of an extensive hemipelvectomy defect [23]. We used this pedicled fillet flap in the reconstruction for our patient based on the following reasoning: the defect in this case was too extensive to be covered using the above-mentioned free flaps; because his left fractured pelvis was complicated by a deep-bone infection and his left leg was almost completely nonfunctional, hemipelvectomy was unavoidable if our patient was to achieve complete recovery and good ambulatory function; and the defect needed to be covered with a solid flap with sufficient endurance for full weight-bearing if out patient was to wear a prosthesis following the hemipelvectomy. Indeed, no choice other than the chosen procedure (hemipelvectomy and fillet flap from the sacrificed limb) appeared able to resolve the aforementioned problems. A possible explanation for our patient’s left lower limb paresis is ischemia of the sciatic nerve as a sequela of TAE, though direct damage in the initial unstable pelvic fracture might also have contributed to this complication, as described by Suzuki *et al.* [10].

Our patient described typical phantom limb pain, and rehabilitation was very challenging for him. However, he returned to his previous occupation 24 months after surgery. Some authors have reported that 68% to 72% of patients who undergo surgery for pelvic fracture return to their jobs, with a mean duration of follow-up of 35 to 47 months [24,25]. In the context of these reports, our case achieved quite satisfactory outcomes.

## Conclusions

Severe peripelvic Morel-Lavallée lesions and gluteal muscle necrosis following TAE can occur simultaneously after unstable pelvic fractures. Physicians should recognize that these entities can result in life-threatening sepsis and, therefore, should attempt to detect them as early as possible. In a case in which hemipelvectomy is unavoidable, the pedicled upper and lower leg in-continuity fillet flap may provide satisfactory outcomes.

## Consent

Written informed consent was obtained from the patient and his family for publication of this case report and any accompanying images. A copy of the written consent is available for review by the Editor-in-Chief of this journal.

## Abbreviations

CT: Computed tomography
MRI: Magnetic resonance imaging
TAE: Transcatheter angiographic embolization
